# A comparison of free-response and multiple-choice questions on the American Board of Pathology primary certification examinations

**DOI:** 10.1016/j.acpath.2026.100248

**Published:** 2026-04-13

**Authors:** Gary W. Procop, Ty McCarthy, Anthony Schlinsog, Mohiedean Ghofrani

**Affiliations:** a American Board of Pathology, Tampa, FL, USA; b PeaceHealth Southwest Medical Center, Vancouver, WA, USA

**Keywords:** Board certification, Free-response questions, Multiple-choice questions

## Abstract

The American Board of Pathology is exploring alternate forms of assessment, and herein has undertaken a comparison between free-response, short-answer questions with paired, construct-equivalent standard multiple-choice, closed-response questions. Four short-answer, free-response questions were compared with four paired multiple-choice, closed-response questions that had equivalent stems and measured the same constructs. The percentage correct responses of the paired question types were compared using chi square analysis, with a level of significance set at *P* < 0.05. Candidates were surveyed regarding the perceived difficulty of the short-answer question type, the question type that they preferred, and why the preferred question type was favored. All four short-answer items had fewer correct responses compared with their paired multiple-choice questions. The percent-correct responses for the short-answer items compared with the paired multiple-choice items, respectively, were: item 1: 73.5% vs 91.6%, item 2: 79.0% vs 90.8%, item 3: 3.3% vs 28.6%, and item 4: 64.7% vs 90.0%. All differences were statistically significant at *P* < 0.01. The short-answer questions, even when liberally graded, were much more difficult compared with the corresponding multiple-choice questions, even though the questions targeted for this evaluation were simple recall type items. The short-answer item responses raised questions about the clarity of the stem for one item, whereas the response to another item clearly disclosed a lack of understanding concerning the construct tested. The candidate survey regarding these question types disclosed an overwhelming candidate preference for multiple-choice questions.

## Introduction

The American Board of Pathology (ABPath) administers certification examinations in four primary specialties and eleven subspecialties of pathology. These uniformly consist of closed-response (i.e. multiple-choice) items that have been constructed, reviewed, and edited by the relevant subspecialty experts, as well as a medical editor. In 2023, the American Board of Pathology made a resolution to investigate a variety of assessment formats that would broaden the assessment of candidates to more thoroughly assess competency, as well as knowledge.^1^ It has been suggested that free-response (i.e. open-ended) questions better interrogate higher-level cognitive functions compared with standard closed-response (e.g. multiple-choice)-type questions.^2^^,^^3^ To investigate this further, we included a free-response (i.e. open-ended, short answer) question that was paired with a standard multiple-choice question on each of our four primary certification forms in the 2025 Spring administration.

## Materials and methods

Two board-certified pathologists with subspecialty expertise in anatomic and clinical pathology reviewed the completed 2025 anatomic and clinical pathology examination forms, respectively. They chose one question from each of the two forms of the Spring 2025 anatomic pathology examination and one question from each of the two forms of the Spring clinical pathology examination. The questions chosen were written (i.e. recall)-type items, in contrast to practical or virtual microscopy items, which are higher-level questions that require interpretation. The stems of the chosen items were used to construct a free-response question, which was a simple fill-in-the-blank format. The content of the paired item stems was therefore the same so that the paired items assessed the same construct (Table 1). The only appreciable difference was in the response format.

**Table 1:** The multiple choice and corresponding free response items compared.

Item	Multiple choice question	Free response question
AP Form A	What is the most likely diagnosis for a diandric diploid genotype performed on products of conception that exhibited abnormal villous morphology? A. **Complete mole** B. Nonmolar hydropic change C. Partial mole D. Fetal aneuploidy	What is the most likely diagnosis for a diandric diploid genotype performed on products of conception that exhibited abnormal villous morphology? Fill in the blank: __________
AP Form B	Which renal tumor is associated with perinuclear halos and crinkly nuclei? A. Oncocytoma B. Metanephric adenoma C. Medullary carcinoma D. **Chromophobe carcinoma**	Which renal tumor is associated with perinuclear halos and crinkly nuclei? Fill in the blank: __________
CP Form A	In new assay verification of a quantitative molecular amplification test, what is probit analysis used to determine? A. Analytical measurement range B. Commutability C. **Limit of detection** D. Quantitative linearity E. Reproducibility	In new assay verification of a quantitative molecular amplification test, what is probit analysis used to determine? Fill in the blank: __________
CP Form B	What is the binding site for the IgG biphasic autoantibody in paroxysmal cold hemoglobinuria? A. CD55 B. CD59 C. E Antigen D. I Antigen E. **P Antigen**	What is the binding site for the IgG biphasic autoantibody in paroxysmal cold hemoglobinuria? Fill in the blank: __________

AP: anatomic pathology examination; CP: clinical pathology examination.

The boldselection in the multiple-choice question indicates the correct answer. Providing that answer or a reasonable variation thereof, with no penalty for misspelling, resulted in extra credit for the free-response question.

The free-response question was presented at the beginning of the examination. The candidates were informed through a preceding instructional page that the ABPath was investigating this new type of question. It explained that extra credit would be given to candidates who successfully completed the free-response question. It also explained that there was no risk to the candidate's examination score for incorrect responses. The paired multiple-choice item was presented in the subsequent section of the examination. The multiple-choice questions that corresponded with the free-response question were considered “test” questions or questions under evaluation and were not scored. The candidates were electronically prohibited from returning to and modifying the free-response item after viewing the multiple-choice format. A very liberal approach was used for the manual grading of the free-response items, with no points removed for misspellings; points were provided for various presentations of the correct responses (e.g. for the item that concerned a complete mole, both “complete mole” and “complete hydatidiform mole” were considered correct responses). The percentage of free-response questions answered correctly was compared with the paired multiple-choice questions (Table 2). Chi-square analysis was used for statistical comparison.

**Table 2:** Comparison of multiple choice and free response correct responses.

Exam/Form	Number of candidates	MCQ correct (%)	MCQ incorrect (%)	FRQ correct (%)	FRQ incorrect (%)	*P-*value
AP/Form A	309	283 (91.6 %)	26 (8.4 %)	227 (73.5 %)	82 (26.5 %)	<0.01
AP/Form B	305	277 (90.8 %)	28 (9.2 %)	241 (79.0 %)	64 (21.0 %)	<0.01
CP/Form A	276	79 (28.6 %)	197 (71.4 %)	9 (3.3 %)	267 (96.7 %)	<0.01
CP/Form B	269	242 (90.0 %)	27 (10.0 %)	174 (64.7 %)	95 (35.3 %)	<0.01

AP: anatomic pathology; CP: clinical pathology; MCQ: multiple choice question; FRQ: free response question.

The opinions of the candidates were sought concerning the free-response, short-answer-type item as part of the postexamination survey. The candidates were asked to respond, on a Likert scale, to the statement: *The difficulty of the short answer question at the beginning of examination was more challenging to answer than multiple-choice questions* (Table 3). Candidates were also asked which type of question they preferred and were given an opportunity to make comments (Table 3). ChatGPT-4o (Open AI, San Francisco, CA) was used to collate the themes from the candidates’ responses regarding why they preferred a particular question type (Table 4, Table 5). The five most frequent themes are provided.

**Table 3:** Difficulty ratings and item type preference.

Anatomic pathology candidates	The difficulty of the short answer question at the beginning of examination was more challenging to answer than multiple-choice questions.
Strongly agree	121 (20%)
Somewhat agree	115 (19%)
Neutral	197 (33%)
Somewhat disagree	90 (15%)
Strongly disagree	57 (9%)
N/A or don't know	22 (4%)
Total	602 (100%)
	Which of the following question types do you prefer?
Short answer	46 (8%)
Multiple choice	555 (92%)
Clinical pathology candidates	The difficulty of the short answer question at the beginning of examination was more challenging to answer than multiple-choice questions.
Strongly agree	262 (49%)
Somewhat agree	92 (17%)
Neutral	110 (21%)
Somewhat disagree	34 (6%)
Strongly disagree	16 (3%)
N/A or don't know	20 (4%)
Total	534 (100%)
	Which of the following question types do you prefer?
Short answer	38 (7%)
Multiple choice	493 (93%)

**Table 4:** Reasons anatomic pathology candidates favored an item type.^a^

Top five reasons why anatomic pathology candidates Preferred multiple choice questions
1. Ease of recall and memory jogging: Multiple-choice questions help jog memory and provide context clues, especially under high-stress situations like exams. They facilitate recall of complex terms and entities that may be hard to retrieve spontaneously.
2. Efficiency and speed: Multiple-choice questions are faster to answer compared to typing out responses, which is particularly important in a time-limited exam setting. They save time and reduce cognitive fatigue during long testing sessions.
3. Reduced subjectivity and grading ambiguity: Multiple choice formats ensure uniform grading and eliminate issues with varied phrasing, spelling errors, or interpretation discrepancies in short answer responses.
4. Process of elimination: Answer options allow candidates to use logical reasoning to narrow down the correct choice, even when unsure of the exact answer.
5. Familiarity and training: Most medical training and prior exams including USMLE^b^, utilize multiple-choice formats. Candidates are accustomed to this style and often prepare using similar practice question banks.
Top five reasons why anatomic pathology candidates Preferred short answer questions
1. Opportunity to demonstrate thought process and reasoning: Short-answer questions allow candidates to explain their diagnostic process, reasoning, and differentials, which is more aligned with real-world pathology practice.
2. Reduced guesswork and confusion: Multiple-choice questions are often seen as confusing due to tricky wording, distractor answers, or insufficient context, whereas short-answer formats minimize second-guessing and allow candidates to focus on their actual knowledge.
3. Partial credit for knowledge: Short-answer questions provide the opportunity for partial credit, recognizing the breadth of a candidate's knowledge even if their final answer is not completely correct.
4. Alignment with real-world practice: Short-answer questions resemble real-world scenarios in pathology where explanations, reasoning, and additional testing are part of the diagnostic process.
5. Fatigue and anxiety management: Long exams are mentally and physically exhausting, and short-answer formats may reduce the stress and fatigue associated with guessing multiple-choice answers.

a ChatGPT-4o was used to review the candidate responses and determine the top five themes.

b USMLE: United States Medical Licensing Examination.

**Table 5:** Reasons clinical pathology candidates favored an item type.^a^

Top five reasons why clinical pathology candidates Preferred multiple choice questions
1. Efficiency and speed: Multiple choice questions are faster to answer compared to short-answer questions. They save time, especially in a time-constrained exam environment.
2. Reduced stress: Multiple-choice formats are less stressful, as they do not require formulating answers from scratch or worrying about spelling and phrasing.
3. Objective scoring: Multiple-choice questions allow for standardized grading, avoiding subjective interpretation or potential bias in scoring.
4. Process of elimination: Test-takers can use the process of elimination to arrive at the correct answer, which is not possible with open-ended questions.
5. Memory recall support: Seeing answer options helps jog memory and can trigger recognition of the correct answer, making recall easier.
Top five reasons why clinical pathology candidates Preferred short answer questions
1. Expression of thought process and knowledge: Many respondents emphasized that short-answer questions allow them to better demonstrate their thinking process, reasoning, and knowledge base. They felt these questions provides an opportunity to explain their answers and showcase their understanding more effectively than multiple-choice questions.
2. Reduced confusion: Short-answer questions help eliminate the confusion often caused by closely similar answer choices in multiple-choice formats. Respondents noted that they are less likely to second-guess themselves or be tricked by the options provided.
3. Nuanced understanding: Short-answer formats enable candidates to capture the nuances of their thinking, particularly when they are torn between multiple options in a multiple-choice scenario.
4. Straightforward nature: Respondents found short-answer questions to be more direct, straightforward, and less complex compared to multiple-choice questions, which often rely on distractors.
5. Opportunity for partial credit: Short-answer questions may allow examiners to assess partial understanding and award partial credit, which is not possible in multiple-choice formats.

a ChatGPT-4o was used to review the candidate responses and determine the top five themes.

## Results

Three hundred and nine candidates received Form A of the Spring 2025 anatomic pathology primary certification examination, whereas 305 candidates received Form B. Two hundred and seventy-six candidates received Form A of the Spring 2025 clinical pathology primary certification examination, whereas 269 received Form B.

A multiple-choice item on Form A of the anatomic pathology examination A that concerned the diagnosis of a complete hydatidiform mole was selected and compared with an analogous free-response, short-answer item. The correct response on both was “complete mole,” with liberal grading of the short-answer response as described above. For the multiple-choice item, 283/309 candidates (91.6%) answered the item correctly, whereas 227/309 (73.5%) answered the corresponding free-response item correctly. Thirty-two of the 82 candidates who did not answer the free-response question correctly did not attempt to answer the question. The difference in the correct response rate was statistically significant (*P* < 0.01). Several of the answers in the short-answer item responses raised issues concerning the wording of the stem that would not have been available from the multiple-choice question format.

A multiple-choice item on Form B of the anatomic pathology examination that concerned the morphologic features of the chromophobe variant of renal cell carcinoma was selected and compared with an analogous free-response item. For the multiple-choice item, 277/305 candidates (90.8%) answered it correctly, whereas 241/305 (79.0%) answered the corresponding free-response item correctly. Nineteen of the 64 candidates who did not answer the free-response question correctly did not attempt to answer the question. The difference in the correct response rate was statistically significant (*P* < 0.01).

A multiple-choice item on Form A of the clinical pathology examination that concerned the use of probit analysis in the verification of a molecular assay was selected and compared with an analogous free-response item. For the multiple-choice item, 79/276 candidates (28.6%) answered the item correctly, whereas 9/276 (3.3%) answered the corresponding free-response item correctly. Seventy-nine of the 267 candidates who did not answer the free-response question correctly did not attempt to answer the question. The difference in the correct response rate was statistically significant (*P* < 0.01).

A multiple-choice item on Form B of the clinical pathology examination that concerned the cellular binding site in paroxysmal cold hemoglobinuria was selected and compared with an analogous free-response item. For the multiple-choice item, 242/269 candidates (90.0%) answered correctly, whereas 174/269 (64.7%) answered the free-response item correctly. Twenty-one of the 95 candidates who did not answer the free-response question correctly did not attempt to answer the question. The difference in the correct response rate was statistically significant (*P* < 0.01).

The responses to the postsurvey questions are provided (Table 3) as are the large language model-collated top five themes concerning why candidates favored a particular item type (Table 4, Table 5).

## Discussion

The American Board of Pathology is committed to evaluating a wide variety of assessment strategies that assess knowledge, skills, behaviors, and competencies more effectively. The three types of items (i.e. questions)–written, practical, and virtual microscopy–are used selectively on certification examinations, and all have a closed-response, multiple-choice format. Although much has been written about the performance of free-response questions versus multiple-choice questions, we thought it prudent to determine if differences exist in the ability of the candidates assessed by ABPath to answer free-response items compared with multiple-choice questions.

We used stem-equivalence to maintain construct equivalence in this assessment, which has been suggested as a valid approach.^4^^,^^5^ The items selected for comparison were simple written-recall-type items, which did not require complex interpretations (i.e. either the candidate knew the correct response or they did not). Otherwise stated, a single construct was tested (i.e. knowledge), in contrast to a practical-type item, for example, that interrogates both knowledge and interpretive ability (i.e. does the candidate know what is being shown, and can they answer the associated question?).

There was a concern that if the multiple-choice question was presented first that it would cue the candidate to answer the free-response question correctly. Therefore, the free-response question was presented first with the analogous multiple-choice item presented in the next section of the examination. This format also prohibited the candidate from returning to the free-response item and changing their answer after viewing the multiple-choice format, which ensured a truer comparison of these item types.

The correct answers for multiple-choice items were consistently greater than those for the corresponding free-response items, suggesting that multiple-choice items do not interrogate the knowledge of the candidate as well as free-response items. This finding is supported by numerous studies. Breuer et al. performed a meta-analysis of 102 primary studies that examined a variety of free-response items (e.g. short-answer, essay) versus close-ended assessment items (e.g. multiple choice, true-false).^6^ They employed multilevel random-effects models to estimate the mean differences between the open-ended and close-ended scores, as well as the correlations between these scores. They found that close-ended exams produced consistently higher scores than open-ended examinations, and that this effect was consistent across different assessment domains (e.g. reading/writing, quantitative ability, etc.). They, however, described a good correlation between open-ended and close-ended assessments (r = 0.67) and suggested that these approaches measure related, but not identical, constructs. The greatest correlation was in the assessment of quantitative knowledge (r = 0.85).

Closed-response questions, like multiple-choice questions, are the favored item type for many examinations for a variety of reasons. The objectivity, economy, and efficiency of scoring closed-response items make them an important and convenient tool for the assessment of a wide variety of content areas. The more items that can be assessed within a given time frame increases the reliability of the examination. Conversely, closed-response formats are susceptible to construct-irrelevant test-taking strategies such as guessing and other strategies of the test-wise candidate.^6^ The implication is that closed-response formats tend to inflate performance compared with free-response formats, secondary to guessing and recognition-based processes (i.e. if the correct answer is one among several selections, then the visualization of that selection can incite memory retrieval).

Not surprisingly, according to the survey results, the candidates overwhelmingly preferred the multiple-choice format to the short-answer, free-response format. Test takers viewed closed-ended question formats as easier, which has been described by others.^7^ The candidates who preferred the multiple-choice format cite using the “process of elimination” and stimulated memory recall (i.e. seeing the answers among the choices) as reasons for their preference (Table 4, Table 5). It has been suggested that closed-ended questions are more likely to demonstrate recall or recognition ability rather than a true understanding of the content, which is consistent with our findings.^8^

As, noted, free-response, open-ended formats are thought to interrogate higher-level cognitive processes that incorporate judgment.^2^^,^^3^ The disadvantages of open-ended formats include the effort, complexity, and time to complete, which limits the number of items on an assessment, and the potential subjectivity of scoring.^9^ Recent reports, however, suggest that artificial intelligence large language models may be able to significantly assist with the grading of free-response questions, as well as even more complex item types.^10^

There are some advanced closed-response items such as coupled-multiple response items that have been reported to produce results that are similar to results from free-response items; these, however, can be very challenging to construct.^11^ Others have suggested that multiple-choice questions may be relatively compatible with student performance on free-response questions if the answers from the multiple-choice questions are graded in a weighted manner (i.e. assigning partial credit) but this is also an added complexity.^12^

This study supports previous findings that suggest that closed-response, multiple-choice examination items are easier for candidates and that scores based on multiple-choice examinations are comparatively inflated. There is, however, a practical tradeoff that includes the number of items that can reasonably be administered and the ease of grading multiple-choice items. Free-response items are likely best employed when it is important to ensure that a complex concept is thoroughly understood. Rudolph et al.*,* when discussing best practices in examination construction, advise that “instructors should select item types that align with the intended learning objectives to be measured on the examination.”^13^

The ABPath is committed to developing assessments for knowledge, competency, and professional behavior. The strength of multiple-choice questions, which is the item type currently used by ABPath, is their ability to cover a vast amount of material in a timely and efficient manner. Unfortunately, this item type is also susceptible to “gaming” by test-wise candidates.^14^^,^^15^ Therefore, in addition to initiating pilot projects to assess competency, the ABPath also undertook this knowledge assessment pilot using two different item types of recall (i.e. not interpretive)-type questions. These findings, which are supported by similar findings in the educational literature, demonstrated that free-response items, although more challenging to grade, better interrogate the knowledge of the candidate compared with multiple-choice questions.^2^^,^^3^ There are many different assessment strategies and many different items types. Leaders in the field of assessment support using assessment strategies that are best suited to evaluate the construct under evaluation.^16^ Although the trustees of the ABPath have yet to determine how or if free-response items will be used in future assessments, they are better informed about the strengths and limitations of these different item types when considering future assessment strategies.

## Declaration of competing interest

There are no conflicts of interest for any of the authors of this manuscript.
